# Immunohistochemical study of pig retinal development

**Published:** 2009-09-21

**Authors:** Jasenka Guduric-Fuchs, Laura J. Ringland, Ping Gu, Margaret Dellett, Desmond B. Archer, Tiziana Cogliati

**Affiliations:** Centre for Vision and Vascular Sciences, Queen’s University Belfast, United Kingdom

## Abstract

**Purpose:**

The pig eye is similar to the human eye in terms of anatomy, vasculature, and photoreceptor distribution, and therefore provides an attractive animal model for research into retinal disease. The purpose of this study was to characterize retinal histology in the developing and mature pig retina using antibodies to well established retinal cell markers commonly used in rodents.

**Methods:**

Eyes were enucleated from fetuses in the 9th week of gestation, 1 week old piglets and 6 months old adult animals. Eyeglobes were fixed and cryosectioned. A panel of antibodies to well established retinal markers was employed for immunohistochemistry. Fluorescently labeled secondary antibodies were used for signal detection, and images were acquired by confocal microscopy. Mouse retina at postnatal day (P) 5 was used as a reference for this study to compare progression of histogenesis. Most of the primary antibodies have previously been used on mouse tissue.

**Results:**

Most of the studied markers were detected in midgestation pig retina, and the majority had a similar distribution in pig as in P5 mouse retina. However, rhodopsin immunolabeling was detected in pig retina at midgestation but not in P5 mouse retina. Contrary to findings in all rodents, horizontal cells were Islet1-positive and cones were calbindin-immunoreactive in pig retina, as has also been shown for the primate retina. Recoverin and rhodopsin immunolabeling revealed an increase in the length of photoreceptor segments in 6 months, compared to 1 week old animals.

**Conclusions:**

Comparison with the published data on human retina revealed similar marker distribution and histogenesis progression in the pig and human retina, supporting the pig as a valuable animal model for studies on retinal disease and repair. Furthermore, this study provides information about the dynamics of retinal histogenesis in the pig and validates a panel of antibodies that reliably detects developing and mature retinal cell phenotypes in the pig retina.

## Introduction

Retinal cellular inventory and development are generally conserved across many studied vertebrate species. However, a range of histological and functional differences exist between individual species. Among nonprimate mammals, the pig eye most closely resembles the human eye, with similar size and comparable histological and physiologic features. Although the gestation period for pig (112–115 days) is significantly shorter compared to human, both human and pig retina are well developed at birth [1,2]. The architecture of the pig retina comprises an area centralis enriched in cones that resembles the human macula, making it an attractive model for preclinical testing [1,3]

Pig models for retinitis pigmentosa, glaucoma, and retinal detachment have been developed [4-6]. Transgenic pigs with systemic expression of green [7] and red fluorescent proteins [8] have also been produced. Furthermore, due to its size, anatomy, and vasculature, the pig eye has been useful for modeling human ocular surgery [9-11]. Finally, pigs have been used for isolation of retinal progenitor cells, and subretinal allotransplantation of these cells demonstrates their ability to migrate, morphologically differentiate, and express retinal cell markers [12,13]. Similarly, adult retinal stem cells have been isolated and characterized from pig ciliary and iris epithelia [14,15].

Several reported in vitro studies on pig retinal neuronal survival and physiology have used immunohistochemical tools to identify cell phenotypes in the primary retinal cell culture [16-19]. At present, there are limited data on the distribution of immunohistochemical markers in the adult pig retina [20-22] and only few studies on pig retinal development [23,24]. Better understanding of pig retinal development is important to fully exploit this model’s potential for the study of eye diseases. Similarly, appropriate tools to investigate retinal histology in the pig are required to follow stem cell differentiation during development, in vitro, and after transplantation.

The aim of the present study was twofold: 1) to characterize antibodies to retina-specific markers commonly used in rodents as tools for further investigations in the pig; and 2) to conduct an immunohistochemical study of retinal histogenesis in the pig.

## Methods

### Animal models

All animal procedures were performed in compliance with the UK Animals (Scientific procedures) Act 1986. Mixed sex white Landrance pigs were obtained from Agri-Food and Biosciences Institute, Northern Ireland (Large Park, Hillsborough, Co. Down, Northern Ireland, UK) where animals were commercially bred on slats and fed with granulated pig food. The pigs were anaesthetised with intra-muscular azoperone (15 mg/kg) and ketamine (20 mg/kg) and euthanized with intravenous or intra-cardiac euthanasia-grade pentobarbitone (100 mg/kg).

Eyes were collected from embryos in the ninth gestational week (GW9), 1 week old piglets (1W), and 6 months (6M) old animals. Three pigs from each age group were analyzed.

Postnatal day (P) 5 in the mouse was used as control for antibody functionality and comparison of developmental stage. Wild type C57BL6 mice were bred in 12 h dark/light regime with free access to food and water. P5 mice were euthanized by intraperitoneal injection with pentobarbitone. The eyes from three P5 mice from different litters were analyzed. Comparison with adult mouse retina was performed using the data from published literature which is accordingly cited in the results section.

### Preparation of retina for histology and immunohistochemistry

Eyes were enucleated from euthanized pigs, and the cornea and the lens were removed. The eyecups were fixed in 4% paraformaldehyde in PBS (137 mM NaCl, 2.7 mM KCl, 10 mM Na_2_HPO_4_, 2mM KH_2_PO_4_ pH 7.4) for 1–4 h at room temperature. After fixation the specimens were cryoprotected in 10% sucrose for 6 h followed by 30% sucrose overnight. The eyecups were embedded in optimal cutting temperature compound (OCT; Sakura, Kobe, Japan) and snap frozen in an isopentane bath on dry ice. Transverse 16 µm cryosections were cut, mounted onto Superfrost Plus glass slides (Fisher Scientific, Loughborough, UK) and stored at −80 °C until use.

Slides were thawed at room temperature and post-fixed in 4% formaldehyde (Sigma-Aldrich, Poole, UK) in PBS for 20 min at room temperature. After rinsing in PBS, sections were blocked for 1 h in 10% normal goat serum, 0.3% Triton X-100, 0.01% NaN_3_ in PBS, at room temperature. Slides were rinsed in PBS and incubated for 24 h at 4 °C with primary antibody diluted in 10% normal goat serum, 0.3% Triton X-100, 0.01% NaN_3_ in PBS. The source information and the dilutions for all primary antibodies used are listed in the Table 1. Following removal of the primary antibody, slides were washed 6 times for 5 min in PBS and incubated for 1 h at room temperature in 1:500 fluorescent-conjugated secondary antibody (Alexa Fluor^488^ or Alexa Fluor^568^ goat anti-mouse or goat anti-rabbit) in PBS. Slides were then washed 3 times for 5 min in PBS at room temperature and cell nuclei were counterstained with 10 µg/ml propidium iodide (Sigma-Aldrich, Poole, UK) in PBS containing 10 µg/ml RNase A (Sigma-Aldrich) for 30 min at room temperature, or with 5 μM DAPI (Invitrogen, Paisley, UK) for 10 min. Slides were mounted in fluorescent mounting medium (Dako, Ely, UK). Negative immunohistochemistry (IHC) controls were performed in parallel by omission of primary antibody. Immunoreactive cells were visualized and images recorded with an inverted confocal microscope (Nikon, Model Eclipse TE 2000-U, Tokyo, Japan) using the Nikon Confocal Microscope EZ-C1 software. Representative images were taken from the midperipheral region of the retina.

**Table 1:** Primary antibodies used for immunohistochemical analysis of the pig retina.

**Antibody**	**Cell specificity**	**Host**	**Dilution**	**Source**	**Reference**
Nestin	neural stem/progenitor cells, Müller glia	mouse	1:400	BD Biosciences	[25,41,44]
Pax6	retinal progenitors, ganglion, amacrine, and horizontal cells	rabbit	1:1,000	Chemicon	[28,45]
Ki67	proliferating cells	mouse	1:300	BD Biosciences	[46]
Brn3a	ganglion cells	mouse	1:40	Chemicon	[47]
Neurofilament (NF)-160	ganglion, horizontal, and bipolar cells	mouse	1:350	Sigma	[22,38,48,49]
AP2α	amacrine cells	mouse	1:50	Developmental Studies Hybridoma Bank	[26]
Calretinin	ganglion, amacrine, horizontal, cone photoreceptor, and bipolar cells	mouse	1:500	Chemicon	[33,50]
Calbindin	horizontal, ganglion, amacrine, bipolar, and cone photoreceptor cells	rabbit	1:1,500	Chemicon	[27,51,52]
GAD65	GABAergic amacrine, rare horizontal ganglion cells	mouse	1:1,000	Developmental Studies Hybridoma Bank	[27,28]
Islet1	amacrine, bipolar, ganglion, and horizontal cells	mouse	1:500	Developmental Studies Hybridoma Bank	[29,37]
PKCα	rod bipolar, amacrine, and ganglion cells, cones	mouse	1:400	Sigma	[27,30,53]
Recoverin	photoreceptors, cone bipolar cells, rare ganglion cells	rabbit	1:1,000	K. Koch	[42,51,54]
Rhodopsin (Rho4D2)	rods	mouse	1:100	R. Molday	[2,36,43]
GNAT2	cones	rabbit	1:500	Santa Cruz Biotechnology	[55-57]
P75 neurotrophin receptor	Müller glia and ganglion cells	rabbit	1:350	Promega	[31,32,58]
Glutamine synthetase	Müller glia	mouse	1:500	BD Biosciences	[35,59]
GFAP	astrocytes	rabbit	1:500	DAKO	[60-62]

The cell specificity is described according to the published literature (listed in the reference column) on the antibodies to the same proteins. Details for the primary antibodies (host, dilution used and the source) used in our study are presented.

## Results

### Progenitor cells in the developing pig retina

**Figure 1:**
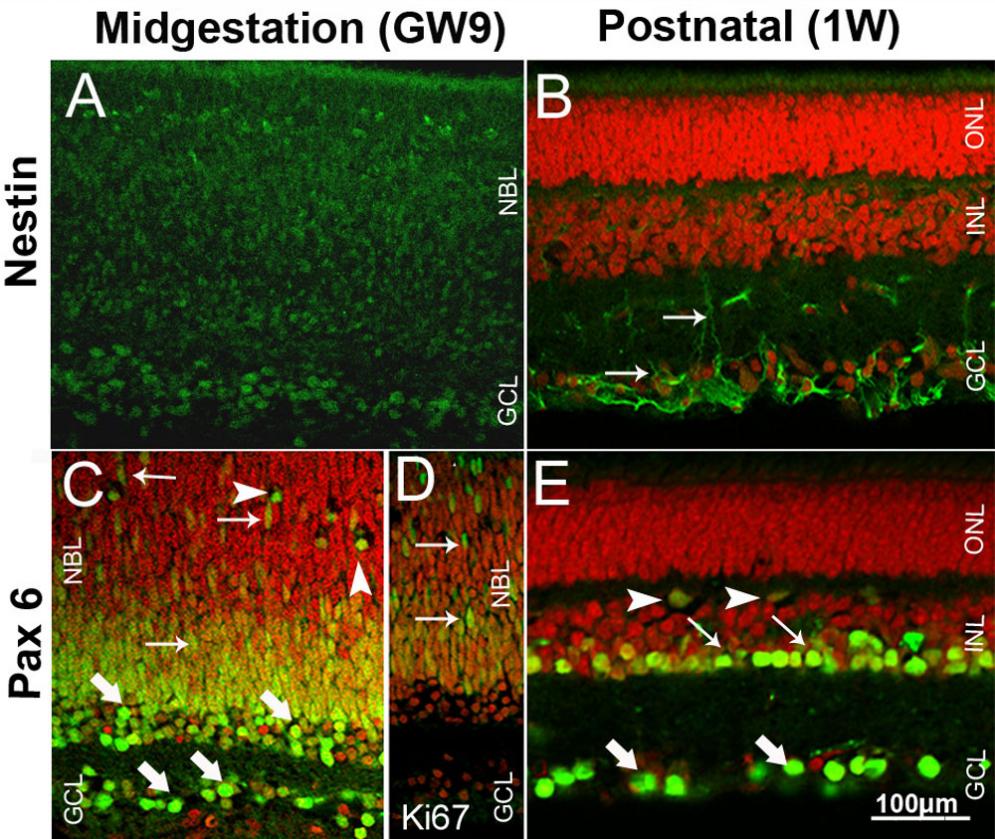
Pig retinal cryosections labeled immunohistochemicaly for nestin, Pax6 and Ki67. **A**: Nestin immunoreactivity is evident throughout GW9 retina. **B**: Nestin positive fibers are in the GCL and IPL (arrows) in 1W retina. **C**: Cells in the GCL, differentiating amacrine (thick arrows), horizontal (arrowheads) and putative retinal progenitors (thin arrows) are Pax6-positive in GW9 retina. **D**: Immunoreactivity for the mitotic marker Ki67 in GW9 retina reveals distribution of progenitor cells similar to that labeled for Pax6 (thin arrows). **E**: Cells in the GCL (thick arrows), amacrine (thin arrows) and some horizontal cells (arrowheads) are Pax6-positive in 1W retina. Nuclei are labeled with PI (red).

**Figure 2:**
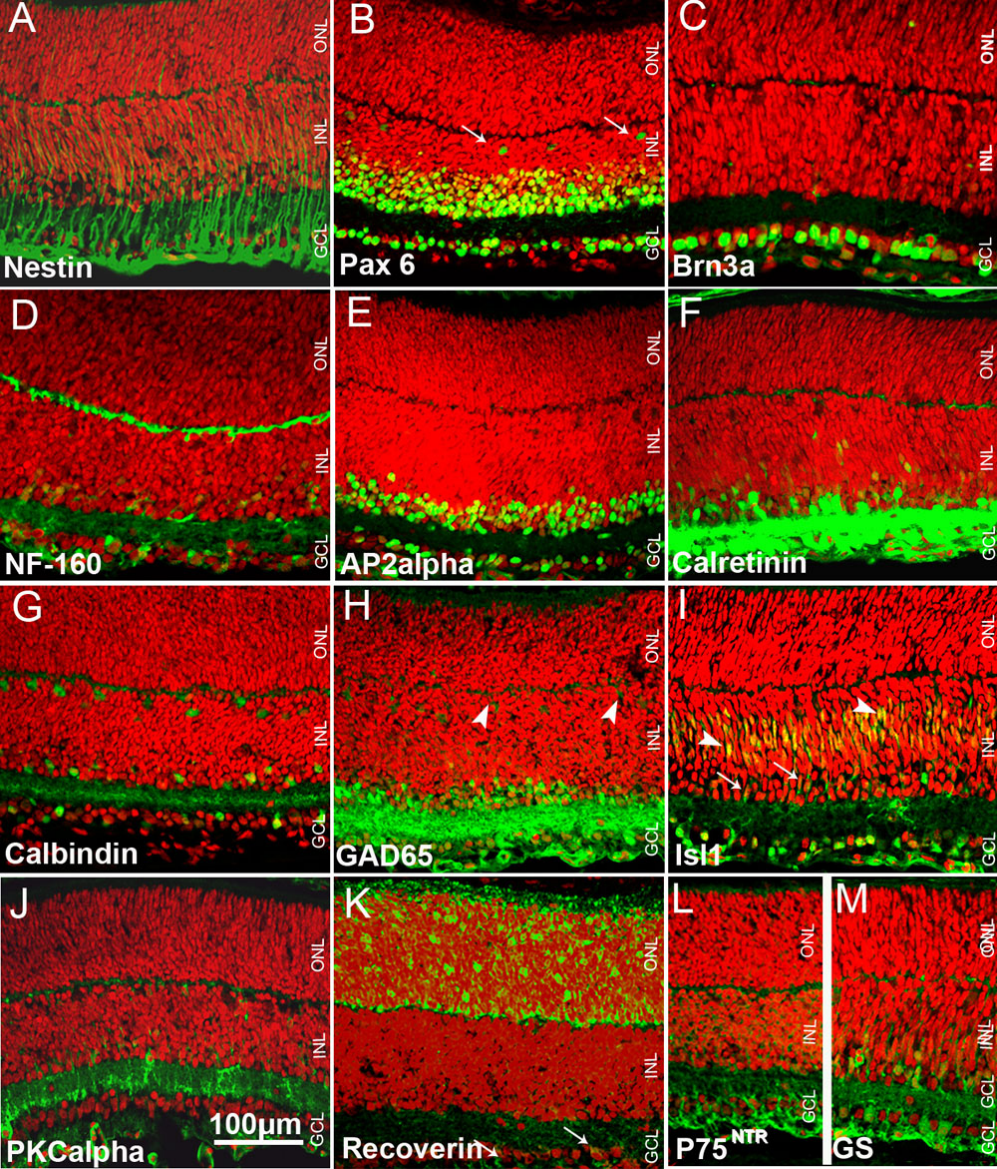
P5 mouse retinal sections labeled immunohistochemicaly. **A**: Radial processes are positive for nestin. **B**: Ganglion, amacrine and horizontal cells (arrows) are Pax6- positive. **C**: Ganglion cells are labeled for Brn3a. **D**: GCL, amacrine cells, IPL, and OPL are NF-160-positive. **E**: Displaced amacrine and amacrine cells are AP2α-positive. **F**: GCL, IPL, amacrine cells and OPL are labeled for calretinin. **G**: Ganglion, amacrine and horizontal cells are calbindin-positive. **H**: IPL, ganglion, amacrine and horizontal cells (arrowheads) are labeled for GAD65. **I**: Ganglion, amacrine (arrows) and bipolar cells (arrowheads) are Islet1-positive. **J**: GCL, IPL, INL and OPL are PKCα-positive. **K**: ONL and some cells in the GCL (arrows) are positive for recoverin. Neurofiber layer, INL and OPL are labeled for P75^NTR^ (**L**) and GS (**M**). Nuclei are labeled with PI (red).

Antibodies to intermediate filament protein nestin and the homeodomain transcription factor Pax6 were used to identify retinal progenitor cells in the developing pig retina (Figure 1). Nestin immunoreactivity was detected throughout GW9 pig retina, reflecting the undifferentiated state of the majority of cells at this stage (Figure 1A). The staining pattern was similar to that found in the P5 mouse retina (Figure 2A), where the strongest immunoreactivity localized to fibers in the ganglion cell layer (GCL). In the postnatal pig retina, nestin immunolabeling was seen in the cell fibers in the GCL and inner plexiform layer (IPL; Figure 1B) probably representing the extensions of Müller glia cells as has been confirmed in the 8W rat retina [25].

In GW9 pig retina, Pax6 strongly labeled most cells in the GCL, cells in the inner portion of the neuroblast layer (NBL) and some cells in the prospective outer plexiform layer (OPL) (Figure 1C). The rounded cells in the inner portion of the NBL represented putative differentiating amacrine cells. Labeled cells with elongated shape and weaker staining found throughout the outer NBL appeared to be migrating progenitors. This observation was corroborated by immunolabeling for Ki67, a marker for proliferating cells, which revealed many mitotic cells throughout the NBL, excluding the innermost portion where amacrine cells differentiate (Figure 1D). Rounded Pax6-labeled cells in the developing OPL (Figure 1C) are suggestive of differentiating horizontal cells [23]. GW9 pig retina contained more migrating progenitor cells compared to P5 mouse retina in which Pax6 labeling mainly colocalized with the prospective position of ganglion, amacrine, and horizontal cells (Figure 2B). Pax6 immunoreactivity was found in ganglion, amacrine, and some horizontal cells in the 1W pig retina (Figure 1E).

### Neurons of the ganglion cell layer

**Figure 3:**
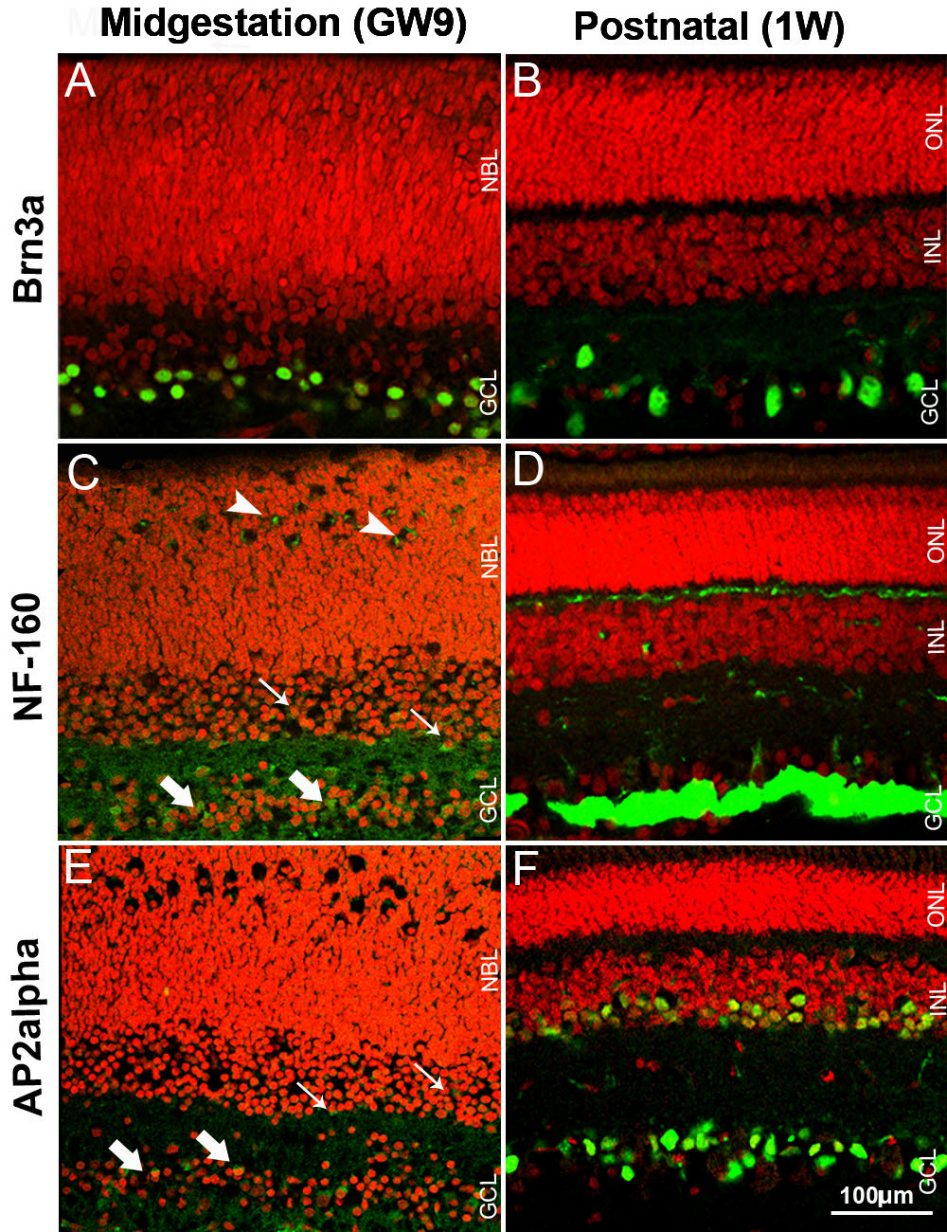
Pig retinal cryosections labeled immunohistochemicaly for the GCL protein markers. **A**: Only ganglion cells are labeled for Brn3a in GW9 retina. **B**: Ganglion cells are Brn3a-positive in 1W retina. **C**: Cells in the GCL (thick arrows), IPL, some developing amacrine (thin arrows) and horizontal cells (arrowheads) are positive for NF-160 in GW9 retina. **D**: GCL, neurofiber layer, and processes in IPL and OPL are NF-160-positive in 1W retina. **E**: Weak labeling for AP2α in the GCL (thick arrows) and prospective INL (thin arrows) is present in GW9 retina **F**: Cells in the GCL and amacrine cells in the INL are APα2-positive in 1W retina. Nuclei are labeled with PI (red).

The antibodies to POU domain transcription factor Brn3a and intermediate neurofilament (NF)-160 were chosen to characterize ganglion cells in the developing, postnatal and mature pig retina (Figure 3). The activating protein α (AP2α) transcription factor, an exclusive marker for amacrine cells, was used to visualize the population of displaced amacrine cells within the GCL. Essentially, the same labeling of the GCL was observed in 1W and 6M pig retinas, therefore only images of 1W pig retinas are shown.

Brn3a immunoreactivity was found exclusively in the GCL in GW9 pig retina (Figure 3A). The observed staining pattern, with densely labeled cells, closely resembled that found in P5 mouse retina (Figure 2C). Brn3a-immunopositive cells were evenly spaced in the GCL of 1W pig retina (Figure 3B).

In GW9 pig retina, NF-160 staining was observed in the GCL, IPL, and in some differentiating amacrine and horizontal cells (Figure 3C). A similar pattern was found in the P5 mouse (Figure 2D), with the exception of strong staining in the OPL, suggestive of more advanced histogenesis. In the 1W pig retina, robust immunoreactivity was seen in the GCL and neurofiber layer, with fine labeling of processes in the IPL and OPL (Figure 3D).

A weak AP2α-positive signal was found in sparse cells in the GCL of GW9 pig retina (Figure 3E). In P5 mouse retina AP2α immunoreactivity was significantly stronger, labeling a large proportion of cells in the GCL and prospective inner nuclear layer (INL) (Figure 2E). Similarly to the staining pattern in adult mouse [26], AP2α in the 1W (Figure 3F) and 6M (not shown) pig retina labeled cells densely distributed in the GCL and the inner portion of the INL, suggestive of displaced amacrine and amacrine cells respectively.

### Neurons of the inner nuclear layer

**Figure 4:**
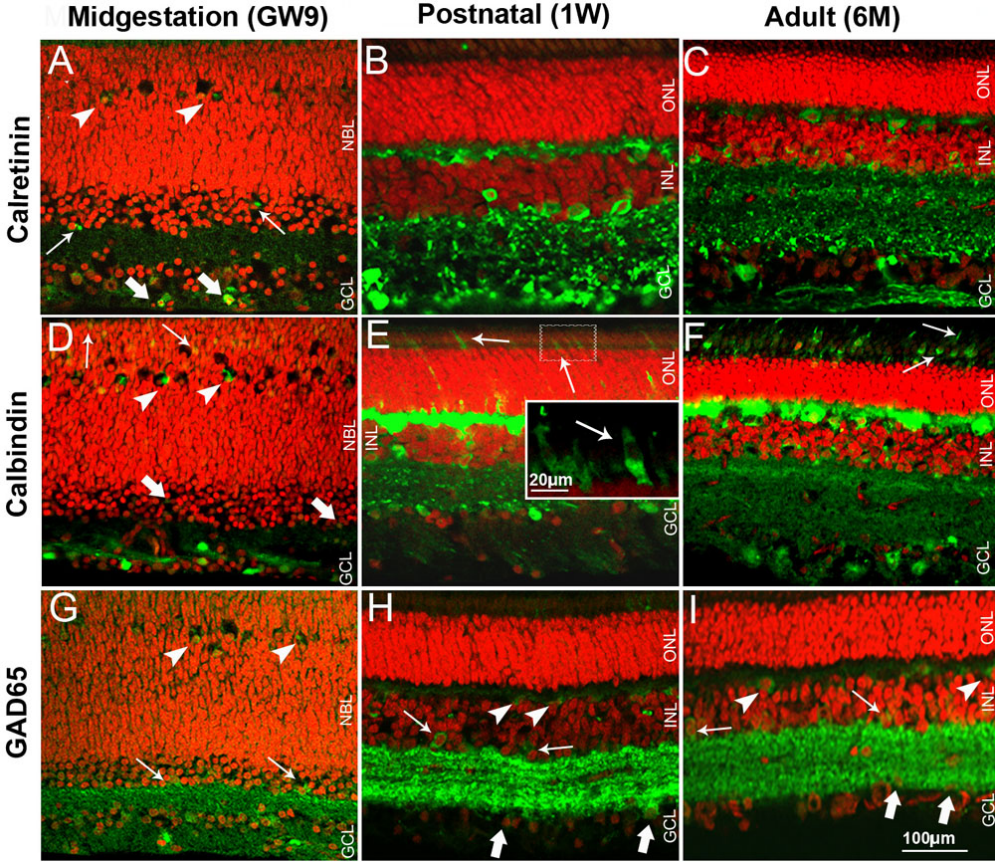
Pig retinal cryosections labeled immunohistochemicaly for calretinin, calbindin, and GAD65. **A**: Ganglion (thick arrows), amacrine (thin arrows) and horizontal cells (arrowheads) are calretinin-labeled in GW9 retina. GCL, IPL, amacrine and horizontal cells are calretinin-positive in 1W retina (**B**) and 6M (**C**) retina. Cells in the GCL, amacrine (thick arrows), differentiating horizontal (arrowheads), and putative cones (thin arrows) are calbindin-positive in GW9 retina. GCL, IPL, amacrine, horizontal cells, and cones (thin arrows) are calbindin-positive in 1W (**E**) and 6M (**F**) retina. **G**: GCL, IPL, amacrine (arrows) and horizontal cells (arrowheads) are GAD65-positive in GW9 retina. IPL, cells in the GCL (thick arrows), amacrine cells (thin arrows) and OPL (arrowheads) are GAD65-positive in 1W (**H**) and 6M (**I**) retina. Nuclei are labeled with PI (red).

**Figure 5:**
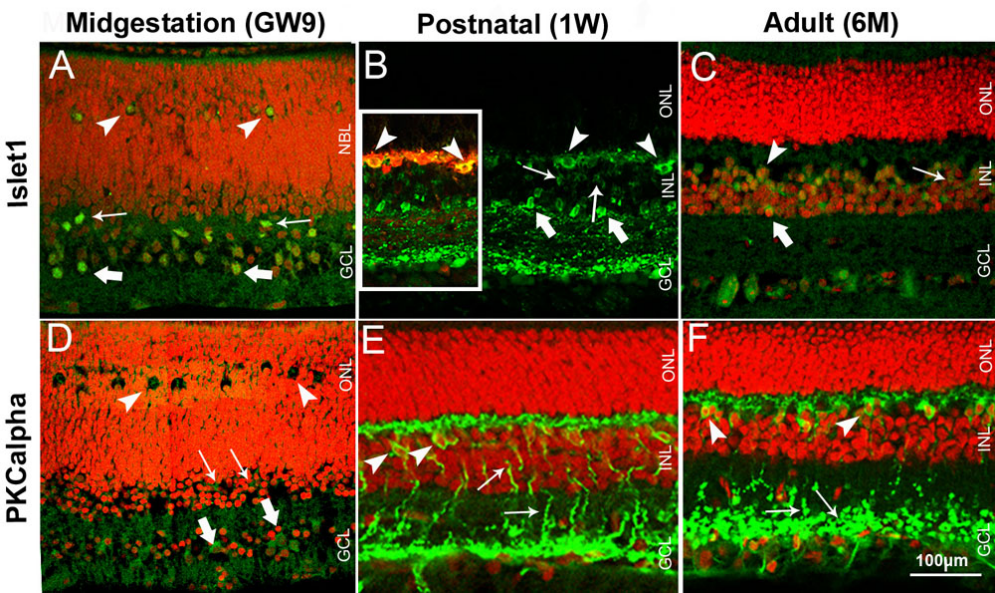
Pig retinal cryosections labeled immunohistochemicaly for Islet1 and PKCα. **A**: Ganglion (thick arrows), amacrine (thin arrows) and horizontal cells (arrowheads) are Islet1-positive in GW9 retina. **B**: Ganglion, amacrine (thick arrows), bipolar (thin arrows) and horizontal cells (arrowheads, also labeled with calbindin in inset **B**) are Islet1-positive in 1W (**B**) and 6M (**C**) retina. **D**: GCL (thick arrows), IPL, prospective INL (thin arrows), and developing OPL (arrowheads) are PKCα-positive in GW9 retina. **E**: Bipolar cell bodies (arrowheads) and their processes (arrows) are PKCα-positive in 1W retina. **F**: Bipolar cell bodies (arrowheads) and axonal endings (arrows) are PKCα-labeled in 6M retina. Nuclei are labeled with PI (red).

To characterize developing neurons in the INL, namely amacrine and horizontal cells, we used antibodies against calretinin, calbindin, and the 65 kDa isoform of glutamic acid decarboxylase (GAD65; Figure 4). Since these markers label cell processes, they also gave a good indication of the development of plexiform layers. The LIM homeodomain transcription factor Islet1 and protein kinase C α isoform (PKCα) were primarily used to visualize bipolar cells (Figure 5).

In the developing pig retina at GW9, calretinin immunoreactivity was found in a subpopulation of cells in the GCL and rare cells in the inner NBL, suggestive of amacrine phenotypes (Figure 4A). Sparse cells in the developing OPL were also weakly labeled. Calretinin immunoreactivity in correspondence to ganglion and amacrine cells was significantly stronger in P5 mouse retina (Figure 2F) than in GW9 pig retina. Weaker labeling was found in mouse OPL. 1W and 6M retina (Figure 4B,C) displayed a calretinin immunostaining pattern similar to that of adult mouse (8–10W), with labeled ganglion, amacrine, and horizontal cells and the distinction of the three strata in the IPL [27].

A subpopulation of cells in the GCL and some cells in the developing OPL were strongly calbindin-immunopositive in GW9 pig retina (Figure 4D). Weak staining was found in sparse developing amacrine cells. Labeling was also seen at the outer edge of the NBL, suggesting immunoreactivity of some developing photoreceptors. Labeling of amacrine cells and of the IPL was more prominent in P5 mouse retina (Figure 2G) compared to GW9 pig, where the INL and IPL only occasionally displayed weak positive signal. The labeling pattern of horizontal, amacrine, and ganglion cells and their processes in the OPL and IPL in both 1W and 6M pig retinas (Figure 4E,F) closely resembled that described for the adult mouse retina. In the pig (1W and 6M) retina, calbindin also labeled sparse photoreceptors, which had a typical wide and tapered cone-like shape (Figure 4E, inset), and did not stain for rhodopsin in double labeling experiments (not shown). Cones appeared immature and mostly inner segments were visualized in 1W pig retina (Figure 4E, inset). At 6M, cones were intensely labeled for calbindin, with inner and outer segments clearly visible (Figure 4F).

In the GW9 pig retina, GAD65 immunostaining was found in amacrine and ganglion cell bodies, with stronger signal localized to the IPL (Figure 4G). Some GAD65-positive cells with immature labeled processes, suggestive of developing horizontal cells, were also highlighted. This staining pattern was similar to P5 mouse retina (Figure 2H), albeit less intense. In mouse, weak signal was also seen in the developing horizontal cells and OPL. In postnatal and adult pig retinas, strong labeling was found in processes layering the IPL, with a characteristic striated appearance (Figure 4H,I) as seen in adult mouse retina [27,28]. Some labeled horizontal cell bodies were visible in 6M pig retina (Figure 4I).

In the GW9 pig retina, moderate to intense Islet1 immunoreactivity was observed in the GCL and in some amacrine cells at the inner border of the NBL (Figure 5A). Strongly immunopositive cells were also localized to the distal part of the NBL in the prospective OPL. The position and round shape of these cells suggests their differentiation toward horizontal cells. Contrary to findings in pig at GW9, no horizontal cells were labeled in P5 mouse retina (Figure 2I). However, developing bipolar cells in the central portion of the INL were strongly Islet1-immunopositive. From the first postnatal week onwards (Figure 5B,C) Islet1-positive staining was found in the GCL, IPL, and INL of the pig retina. At 1W and 6M, Islet1-immunoreactive amacrine cells, as well as densely distributed cell bodies, were visible in the inner and outer portion of the INL, respectively (Figure 5B,C). Some cells in the outer portion of the INL had a spindle shape characteristic of bipolar cells, whereas others appeared rounded, suggestive of horizontal cell phenotypes. Double-labeling experiments with calbindin (Figure 5B, inset) confirmed the presence of Islet1-immunoreactive horizontal cells in 1W pig retina, a feature also described for human retina (12–85 years-old) [29].

Weak PKCα immunolabeling was detected in the GCL, IPL, inner NBL, and in the prospective OPL of GW9 pig retina (Figure 5D). P5 mouse retina displayed a similar labeling pattern (Figure 2J). However, the staining was more prominent compared to GW9 pig. In both 1W and 6M pig retina (Figure 5E,F) oval-shaped immunoreactive cells were found in the INL, with their cell bodies located close to the OPL and their processes extended toward both OPL and IPL, as is characteristic of rod bipolar cells. Axon processes in the IPL were clearly visualized in the 1W pig retina (Figure 5E), whereas staining in the 6M was mainly localized to the axon endings of rod bipolar cells, presented in the form of characteristic swellings or granulate labeling (Figure 5F). Some cell bodies adjacent to the vitreous and their processes were PKCα-immunoreactive in the 1W and 6M pig retinas. No PKCα-positive amacrine or cone photoreceptor cells were detected in the 1W or 6M pig retina, as reported for the mouse (8–10W) [27,30].

### Photoreceptors

**Figure 6:**
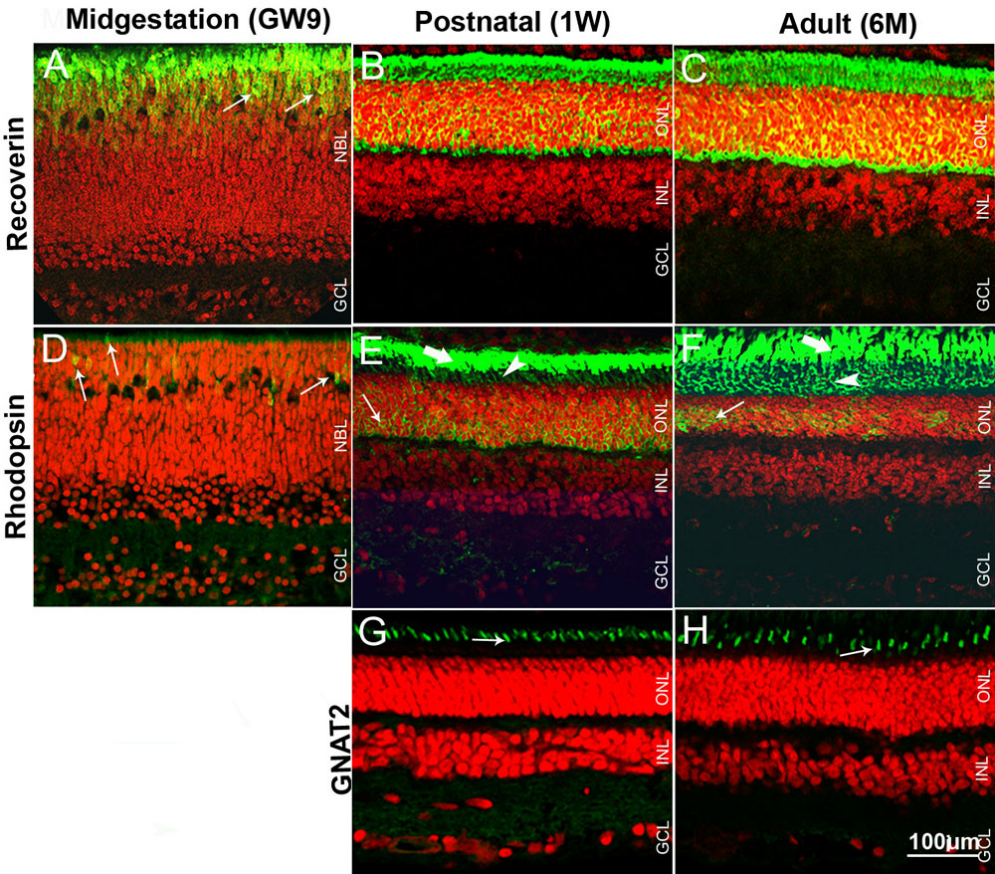
Pig retinal cryosections labeled immunohistochemicaly for photoreceptor protein markers. **A**: Cells in the prospective ONL are positive for recoverin (arrows) in GW9 retina. ONL and photoreceptor segments are recoverin-labeled in 1W (**B**) and 6M (**C**) retina. **D**: Some differentiating rod photoreceptors are positive for rhodopsin in GW9 retina (arrows). **E**: Strong rhodopsin-labeling is present in the outer segments (thick arrows) in 1W retina. Inner segments (arrowheads) and cell bodies (thin arrows) are less intensely stained. **F**: Rhodopsin-positive inner (arrowheads) and outer (thick arrows) segments are more elongated in 6M compared to 1W retina. Some rod cells bodies are also labeled (thin arrows) in 6M retina. Cone outer segments are labeled for GNAT2 in 1W (G) and 6M (**H**) retina (thin arrows). Nuclei are labeled with PI (red).

An antibody to calcium binding protein recoverin, which labels both rods and cones, was used as an early marker of photoreceptor differentiation (Figure 6). Rod photoreceptors were further characterized with an anti-rhodopsin antibody. Antibodies for mouse S-opsin and M-opsin used for this study did not cross-react with pig proteins (data not shown); therefore we used an antibody against Gα subunit of the cone transducin (GNAT2) to label cones in the pig retina.

In the GW9 pig retina, strong recoverin immunoreactivity was detected in putative differentiating photoreceptors, localized to the apical surface of the NBL, and in sparse cells found deeper in the inner NBL (Figure 6A). Recoverin staining was seen throughout the developing ONL in P5 mouse retina (Figure 2K). However, labeling at the apical surface of the NBL was weaker than in GW9 pig. Some recoverin-positive ganglion cells were observed in mouse P5, but not in pi GW9g developing retina. In the 1W pig retina, recoverin immunoreactivity was seen throughout the ONL, including in the inner and outer segments (Figure 6B,C). Photoreceptor segments appeared longer at 6M than at 1W. Some recoverin-positive cells localized to the prospective INL in the GW9 pig retina could be regarded as developing bipolar cells (Figure 6A). However, no conspicuous bipolar cell labeling was observed in 1W or 6M pig retina (Figure 6B,C).

In GW9 pig retina, sparse elongated rhodopsin-immunoreactive cells were observed localized to the prospective ONL, suggestive of early differentiating rod photoreceptors (Figure 6D). Some rudimentary inner segments were also weakly labeled. No rhodopsin-positive cells were detected in the P5 mouse retina with the same antibody (data not shown). Rod outer segments were strongly labeled in both 1W and 6M pig retina, whereas inner segments and some cells bodies displayed weaker staining (Figure 6E,F). Rod outer segments appeared longer and more defined in 6M compared to 1W old animals, suggesting that outer segments continue to extend in length well into the postnatal period.

No labeling with GNAT2 antibody was observed in the GW9 pig retina. Similarly, mouse cones at P5 were not positive for this marker (not shown). Cone outer segments in both 1W and 6M pig retina were strongly immunoreactive for GNAT2 (Figure 6G,H). The size of the cone outer segments slightly increased from the 1W to the 6M pig retina.

### Retinal glial cells

**Figure 7:**
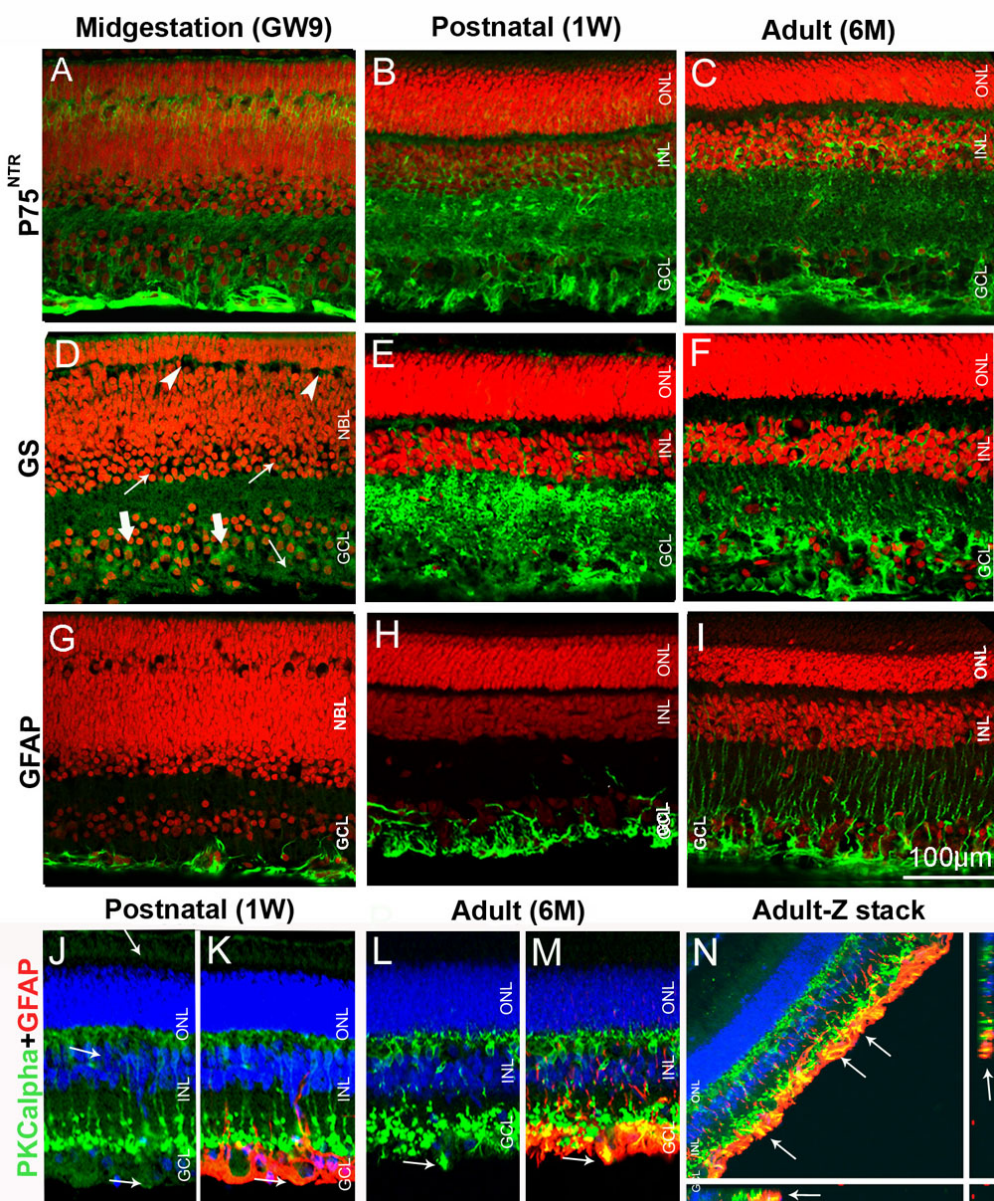
Pig retinal cryosections labeled for glial protein markers. **A**: Processes in the neurofiber layer, GCL, OPL, inner NBL, and developing OPL are P75^NTR^-positive in GW9 retina. Processes in the GCL, IPL, and INL are P75^NTR^-positive in 1W (**B**) and 6M (**C**) retina. **D**: Processes in the GCL (thick arrows), NBL (thin arrows), and developing OPL (arrowheads) are labeled for GS in GW9 retina. GS immunolabeling is present in the GCL, IPL, and INL in 1W (**E**) and 6M (**F**) retina. **G**: Immature astrocytes are GFAP-positive in GW9 retina. **H**: Processes in neurofiber and GCL are GFAP- positive in 1W retina. **I**: Elongated GFAP-positive processes in 6M retina. **J-M**: GFAP-immunopositive astrocytes are PKCα-positive, weakly in 1W (**J, K**, arrows) but strongly in 6M retina (**L, M**, arrows). **N**: A Z-stack image shows extensive double GFAP-PKCα labeling at 6M (arrows). X- and Y- planes are shown to bottom and to the right of the image.

The antibodies to low affinity neurotrophin receptor P75 (P75^NTR^) and glutamine synthetase (GS) were used to characterize Müller glia, whereas an anti-GFAP antibody was used to label astrocytes. In the GW9 pig retina, strong P75^NTR^ staining was present in the neurofiber layer (Figure 7A), similar to P5 mouse retina (Figure 2L). Labeled processes extended through the GCL and IPL and entered the inner portion of the NBL. Additional immunoreactivity was seen in the developing OPL, with the majority of stained cell bodies localized in the distal half of the prospective INL and some cell processes extending to the ONL. From the postnatal period onward the labeling pattern for P75^NTR^ in the pig retina remained unchanged, although it appeared that IPL displayed stronger staining in the 1W compared to the 6M retina (Figure 7B,C).

In the GW9 pig retina GS positive labeling was seen mainly in the GCL, where it likely represents processes of the developing Müller glial cells. Weaker staining was also observed in the IPL and the inner portion of the NBL (Figure 7D). Interestingly P75^NTR^ and GS staining pattern were almost identical in the P5 mouse retina (Figure 2L,M), whereas in the GW9 pig, P75^NTR^ staining was significantly stronger, suggesting temporal advantage in the expression of this marker. GS immunolabeling pattern in the 1W and 6M pig retina was very similar to that seen for P75^NTR^, revealing strong staining in the GCL, IPL and INL, suggestive of Müller glia cells, as it has also been shown for adult (8–10W) rat retina (Figure 7E,F) [31,32]. As seen also for P75^NTR^, somewhat stronger staining for GS was found in the IPL of 1W compared to the 6M pig retina.

In GW9 pig retina, GFAP immunoreactivity was observed adjacent to the vitreous in cells with thin and short processes, suggestive of immature developing astrocytes (Figure 7G). In the 1W retina, the majority of the staining was localized in the neurofiber layer and GCL, with rare processes protruding into the IPL (Figure 7H). Astrocytic processes in the adult retina became denser and longer, extending from the neurofiber layer through the GCL, IPL and INL, occasionally reaching the ONL (Figure 7I). PKCα colocalized with glial fibrillary acidic protein (GFAP) immunostaining in double labeling experiments, identifying PKCα-positive astrocytes (Figure 7J-N). Astrocytes in 1W pig retina were weakly labeled for PKCα, but in 6M pig retina these cells were strongly PKCα immunoreactive (Figure 7J-N).

## Discussion

We have conducted an immunohistochemical study of the pig midgestation, postnatal, and adult retina to establish the timing and progression of retinal development and define markers that reliably identify specific cell types in the pig. The majority of the studied markers were expressed at midgestation, and labeled cells were arranged in a laminar pattern according to their distribution in the mature retina. Nestin and Pax6 immunolabeling revealed a relatively large population of retinal progenitor cells at midgestation. Based on histology and retinal architecture, development appeared less advanced in GW9 pig compared to P5 mouse retina, as suggested by the well demarcated developing OPL present in mouse but not visible in the pig retina. Histogenesis, as detected by immunohistochemistry, appeared to follow a different sequential order in pig compared to mouse. Immunoreactivity of ganglion cells in GW9 pig was similar to that in P5 mouse. Horizontal cells were strongly calbindin- and NF-160-immunoreactive in both GW9 pig and P5 mouse retina. However, AP2α, calretinin, calbindin, Islet1, and PKCα immunolabeling indicated that development of amacrine and bipolar cells was significantly less advanced in GW9 pig compared to P5 mouse. On the contrary, sparse developing rod photoreceptors were identified in GW9 pig retina, while P5 mouse retina did not show any rhodopsin immunoreactivity. These observations suggest that rod differentiation occurs earlier in pig than in mouse, relative to the development of the inner retina.

Bearing in mind the potential of the pig as a preclinical animal model for retinal disease, it is important to highlight the similarities and differences between human and pig retina. Similar to the human, the pig retina is relatively mature at birth with all layers and retinal cell types present in one-week-old animals. GW9 pig retinas appeared to be at an earlier developmental stage than GW20–21 human retina, based on published literature [33]. At GW9 in pig only early signs of OPL development were evident, whereas in human GW9 retina separation between ONL and INL is fully accomplished [33]. Pax6 labeling in GW9 pig retina revealed cells of elongated shape distributed throughout the NBL, likely migrating retinal progenitors, whereas Pax6 immunostaining has been described in the human retina at GW21 only in differentiated ganglion cells and differentiating amacrine and horizontal cells [34]. Similarly, expression of the calcium binding proteins, calretinin and calbindin, appeared to be lagging behind in GW9 pig compared to the GW20–21 human retina. In the GW21 human retina, both calretinin and calbindin robustly label ganglion and amacrine cells, with weaker staining of horizontal cells and additional labeling of cones [33]. However, only sparse cells in the GCL and NBL were calretinin- or calbindin-immunoreactive at GW9 in pig. Additionally, Müller glial cells are strongly GS positive in the GW20 human retina [35], but only weak labeling of the processes in the GCL was seen in the GW9 pig. Early immunolabeling of rhodopsin at GW9 in the pig retina is comparable to that in human, where rhodopsin immunoreactivity has been shown at GW15 in rod inner segments [36]. Although recoverin has been detected in bipolar cells in both mouse and human [27,29], only photoreceptors were labeled with anti-recoverin antibody in the pig retina.

Several antibodies labeled the same cell types in pig and human retina, but displayed different cell specificity in mouse. As in adult human retina (12–85 years old) [29], but contrary to adult mouse (10W) [37], horizontal cells in the pig retina were immunoreactive for Islet1 [29]. Moreover, as in postnatal (4 months-old) and adult (35 years-old) human retina [33], calbindin immunoreactive cones were found in 1W and 6M pig, but not in adult 8–10 week-old mouse retina [26]. However, the antibody against NF-160 subunit labeled horizontal cells in pig (GW9, 1W, 6M) as well as in mouse retina [38], but it has only been reported in retinal ganglion cells in the human retina [22,39,40].

The majority of investigated markers showed the same pattern of immunolabeling in both 1W and 6M pig retina. However, differences were found suggesting that some degree of retinal maturation occurs during the postnatal period. The same is true for the human retina, which reaches maturity by 5 years of age [2]. For example, photoreceptor outer segments continue to grow in length well into the postnatal period in pig as in human retina. Furthermore, GFAP immunolabeling revealed a significant increase in the length of astrocytic processes from 1W to the 6M pig retina. PKCα immunostaining was distributed along the axons of bipolar cells in the 1W pig retina and concentrated at the synaptic site adjacent to the GCL in the 6M retina. Finally, expression of PKCα in astrocytes was stronger in adult compared to 1W pig retina.

The data presented herein should prove useful in future investigations that use pigs as a preclinical model system for retinal disease and repair. Similarity with human retina makes such studies a viable alternative to expensive and ethically problematic experimentation on nonhuman primates.
